# Study protocol for a randomized, blinded, controlled trial of ketamine for acute painful crisis of sickle cell disease

**DOI:** 10.1186/s13063-019-3394-4

**Published:** 2019-05-27

**Authors:** Mohammed S. Alshahrani, Laila Perlas Asonto, Mohamed M. El Tahan, Amal H. Al Sulaibikh, Sukayna Z. Al Faraj, Abdullah A. Al Mulhim, Murad F. Al Abbad, Samar A. Al Nahhash, Moath N. Aldarweesh, Alaa M. Mahmoud, Nisreen Almaghraby, Mohammed A. Al Jumaan, Thamir O. Al Junaid, Faisal M. Al Hawaj, Samar AlKenany, Omaima F. ElSayed, Haitham M. Abdelwahab, Mohamed M. Moussa, Bader K. Alossaimi, Shaikah K. Alotaibi, Talal M. AlMutairi, Duaa A. AlSulaiman, Saad D. Al Shahrani, Donia Alfaraj, Waleed Alhazzani

**Affiliations:** 1 Emergency and Critical Care Departments, King Fahad Hospital of the University, Imam Abdulrahman bin Faisal University-Dammam, AlKhobar, Kingdom of Saudi Arabia; 2 Anesthesia Department, King Fahad Hospital of the University, Imam Abdulrahman bin Faisal University-Dammam, Kingdom of Saudi Arabia, Mansoura University, Mansoura, Egypt; 3 Emergency Department, King Fahad Hospital of the University, Imam Abdulrahman bin Faisal University-Dammam, AlKhobar, Kingdom of Saudi Arabia; 4 Pharmacy Department, King Fahad Hospital of the University, AlKhobar, Kingdom of Saudi Arabia; 5 Department of Clinical Epidemiology and Biostatistics, McMaster University, Hamilton, Canada

**Keywords:** Acute painful crisis, Ketamine, Randomized control trial, Sickle cell disease

## Abstract

**Background:**

Sickle cell disease (SCD) is an inherited hematological disorder where the shape of red blood cells is altered, resulting in the destruction of red blood cells, anemia, and other complications. SCD is prevalent in the southern and eastern provinces of the Arabian peninsula. The most common complications for individuals with SCD are acute painful episodes that require several doses of intravenous opioids, making pain control for these individuals challenging. Instead of opioids, some studies have suggested that ketamine might be used for pain control in acute pain episodes of individuals with SCD. This study aims to evaluate whether the addition of ketamine to morphine can achieve better pain control, decreasing the number of repeated doses of opiates. We hypothesize that early administration of ketamine would lead to a more rapid improvement in pain score and lower opioid requirements.

**Methods and analysis:**

This study will be a prospective, randomized, concealed, blinded, pragmatic parallel group, controlled trial enrolling adult patients with SCD and acute vaso-occlusive crisis pain. All patients will receive standard analgesic therapy during evaluation. Patients randomized to the treatment arm will receive low-dose ketamine (0.3 mg/kg in 0.9% sodium chloride, 100 ml bag) in addition to standard intravenous hydration, while those in the control group will receive a standard dose of morphine (0.1 mg/kg in 0.9% sodium chloride, 100 ml bag) in addition to the standard intravenous hydration. All healthcare providers will be blinded to the treatment arm. Data will be analyzed according to the intention-to-treat principle. The primary outcome is improvement in pain severity using the Numerical Pain Rating Score.

**Trial registration:**

Clinicaltrials.gov, NCT03431285. Registered on 13 February 2018

**Electronic supplementary material:**

The online version of this article (10.1186/s13063-019-3394-4) contains supplementary material, which is available to authorized users.

## Background and rationale

Sickle cell disease (SCD) is an inherited hematological disorder where the shape of red blood cells is altered into sickle-like cells resulting in the destruction of red blood cells leading to anemia and other hematological complications. SCD is prevalent in the southern and eastern provinces of the Arabian peninsula. Acute painful episodes are the most common complications of the disease process; they result from tissue ischemia due to occlusion of the microcirculation with clusters of sickled red blood cells [1]. These episodes usually involve the long bones or the spine and might also involve other organs. An acute painful crisis can be precipitated by cold exposure, dehydration, infection, hypoxia, acidosis, or hypercarbia, or it may not be related to a specific trigger. This condition exposes the patient to severe pain requiring several emergency department (ED) visits, and is considered the most common cause of hospital admissions for SCD patients. The costs of hospital admission for SCD patients have been investigated and, in the United States between 1989 and 1993, 75,000 admissions per year of SCD patients generated a total cost of $475 million per year [2]. Patients with more than three admissions per year with painful crises were found to be at higher risk of early death [3]. The mainstay of therapy for acute painful crisis is hydration and intravenous (IV) opioid analgesia [4]. Existing evidence supports the use of opioid therapy in treating vaso-occlusive crises (VOCs) [5–9]. Nevertheless, the regimen of pain control is challenging for the emergency physician because the management of acute painful crisis requires several doses of IV opioids accompanied by the fear of known side effects and complications associated with these drugs. A study of SCD patients treated for painful crisis showed that an accumulative dose of IV morphine ranged from 4 mg to 26.7 mg with an average dose of 0.05–0.5 mg/kg during 70% of the visits. Fifty percent of SCD patients were admitted at less than 3 h after ED treatment, while 28% of the discharged patients returned to the ED within 3 days [10]. Moreover, as with other chronic pain patients, SCD patients experience opioid-induced hyperalgesia leading to activation of the *N*-methyl-d-aspartate (NMDA) receptors [1].

Ketamine, a noncompetitive NMDA receptor antagonist, may have the potential to modulate opioid-induced hyperalgesia through impaired sensitization of spinal neurons to nociceptive stimuli, and may therefore reduce neuropathic pain. An extensive search of the literature revealed few reports on low-dose ketamine in the management of acute painful crises in SCD patients. The results of these reports were limited by their retrospective designs and the inclusion of relatively small sample sizes [11–13].

A retrospective study of five children and adolescents receiving low-dose ketamine infusion demonstrated a reduction in pain scores in two patients and a significant reduction in opioid utilization in only one patient [12]. A recent Canadian retrospective study adding intravenous ketamine in the management of nine patients with painful sickle cell crisis showed significant reductions in cumulative morphine consumption (146 ± 16.5 mg/day versus 112 ± 12.2 mg/day) and pain scores [14]. Similar reductions in opioid consumption were observed in a retrospective American study of 30 SCD patients with VOC pain [4]. In 2017, Motov et al. [15] performed a prospective, randomized, double-dummy trial where they investigated the analgesic efficacy and adverse effects of low-dose ketamine either by single IV push or short infusion. The authors found that short infusion was associated with significantly lower rates of unreality feeling and sedation while achieving the same analgesic efficacy of a single IV push.

To the best of our knowledge, there are no previously published large, prospective, randomized controlled trials investigating the impact of adding low-dose ketamine on improving the quality of analgesia in patients with VOC in SCD patients.

## Aims and hypotheses

### Primary hypothesis

We hypothesize that early administration of ketamine in combination with standard acute opiate treatment will achieve a more rapid reduction in pain score defined as an improvement in the Numerical Pain Rating Score (NPRS) of 1.5 points or more compared with the control arm for pain relief in SCD patients with VOC pain.

### Primary efficacy aims

The primary efficacy aim of our study is to validate whether the early use of ketamine for the management of acute sickle cell pain crisis will achieve a more effective reduction in pain severity scores.

### Safety aim

Administration of either morphine or ketamine will adhere to standard practice and should not be considered to pose any special safety issues. Nevertheless, monitoring of patients will be performed for the possibility of ketamine-related side effects, both common (nausea, vomiting, and a mild increase in heart rate and blood pressure) and uncommon (laryngospasm, emergence reactions, and nightmares). There is no reason to expect that the incidence will exceed what is encountered in daily practice [16].

Our clinical research data manager is tasked with compiling and reviewing on a regular basis the accumulating data collected to ensure the continuing safety of current participants and those yet to be enrolled. Once any protocol deviation/violation is identified, the issue shall be forwarded by the data manager to the safety monitoring team to handle the deviation/violation.

### Secondary efficacy aims

Secondary aims will be to decrease the ED length of stay, the cumulative use of opioid during ED stays, the rate of hospital admission (defined as the number of patients who needed hospital admission from the ED due to refractory painful crises as opposed to those who were discharged home from the ED) in both arms, and the development of any known side effects of the drugs used.

## Trial design

This study is a prospective, randomized, concealed, blinded, pragmatic parallel group, controlled trial evaluating the efficacy and safety of ketamine in combination with standard treatment for the management of acute pain crisis among SCD patients.

## Methods

This study protocol is reported in accordance with the SPIRIT statement guidelines to promote study quality and transparent interpretation and reporting of study results [17] (Additional file 1).

### Patient and public involvement

No patients were involved either in the trial design or recruitment or any part of the study methods. Ketamine has been widely used as a pain medication in our hospital with an observed positive response. Saudi Food and Drug Authority (SFDA) approval was obtained prior to the use of this medication in the trial as an off-label indication. All patients recruited signed an informed consent, and a thorough trial procedure description between patient and assessor contact was undertaken.

### Study setting

The trial will take place at the ED of a tertiary academic hospital in the eastern province of Saudi Arabia. All patients, irrespective of allocation, will receive a standard dose of non-narcotic analgesia, within 30 min of physician-patient contact with a single dose of either paracetamol (1 g IV infusion over 30 min) or nonsteroidal anti-inflammatory drugs (NSAIDS; either lornoxicam 8–16 mg IV or diclofenac 75 mg intramuscular injection) since it is not ethical to deprive the patient of any kind of analgesia while waiting for screening, randomization, and study drug preparation. The pain score will be measured at 0 and 30 min, after which the consent procedure and randomization will be initiated for patients meeting these criteria: 1) established diagnosis of the painful crisis; and 2) patients with an NPRS greater than 5.

The infusion preparation will be labeled for the patient with a study number from the computer-generated stratification numbers without any other identifying marks. The study nurse assigned to the patient will administer the study drug. Patients, healthcare providers, and outcome assessors will be blinded to the treatment allocation.

Pain scores will be checked at 0, 30, 60, 90, 120 ,and 180 min after the study drug is given.

If no pain relief is achieved within 30 min of the study drug being given, the treating physician will decide to either resume usual practice with morphine or any equivalent pain medication or make an admission decision within a maximum of 180 min. Monitoring of all patients includes electrocardiography, noninvasive blood pressure, pulse oximetry, and temperature. All patients will receive oxygen supplementation through a face mask or nasal prongs during sleep and when required to maintain oxygen saturation higher than 92%. Lactated Ringer’s or NaCl 0.9 solution will be infused at a rate of 2–3 ml/kg/h. Normothermia will be maintained with warming air-enforced blankets if necessary. No attempt will be made to expedite this process. All patients will be included in the intension-to-treat analysis whether admitted or not.

### Eligibility criteria

We will include patients who signed an informed consent who meet the following criteria: 1) patients who are 18 years of age or older diagnosed with SCD based on sickle cell tests or hemoglobin electrophoresis; 2) SCD patients with acute onset of painful crisis developing within 7 days from study recruitment.

### Exclusion criteria

Patients with the following characteristics will be excluded from the study: 1) pregnancy or breast-feeding; 2) patients with altered mental status; 3) patients with body mass index greater than 40 kg/m^2^; 4) patients with significant neurological disease; 5) patients with seizures; 6) patients with acute head or eye injury; 7) patients with high intracranial pressure; 8) patients with known psychiatric disorders; 9) patients with significant cardiac diseases or arrhythmias; 10) patients with significant pulmonary diseases other than acute chest syndrome; 11) patients with significant renal disease (BUN/creatinine ratio < 25); 12) patients with significant hepatic disease (Child-Pugh class B or C); 13) patients with significant endocrine disease; 14) patients with a known allergy to phencyclidine derivatives, ketamine, or morphine; 15) patients with sepsis or septic shock; 16) patients who require circulatory or ventilatory support; 17) patients with alcohol or drug abuse; 18) patients with chronic pain status unrelated to SCD; 19) patients receiving anticonvulsant or antipsychiatric medications, narcotics, or analgesics other than paracetamol and NSAIDs; or 20) patients with communication barriers.

### Recruitment and consent

All physicians and nurses at the ED will be invited to an in-service trial lecture. Pharmacy staff shall be invited to attend this lecture to be conducted by the principal investigator to encourage screening of patients during their shift for adult patients with SCD who will be admitted to the ED for severe painful crisis management with pain NPRS greater than 5 on a standard range from 0 (no pain) to 10 (worst pain imaginable) who require opioid analgesia determined by the attending physician.

Upon identification of patients, the study investigators shall screen potentially eligible patients with the inclusion/exclusion criteria checklist as per the data collection instrument. If the patient is eligible, study investigators will obtain informed consent and explain potential risks and benefits of receiving study interventions (Additional files 2 and 3).

### Interventions

Patients randomized to the intervention group will receive low-dose ketamine (0.3 mg/kg) in 100 ml normal saline infused over 30 min in addition to a standard IV hydration. Rescue pain medication will then be given to patients based on the discretion of the treating physician. Patients randomized to the control group will receive the standard dose of morphine (0.1 mg/kg) in 100 ml normal saline infused over 30 min in addition to a standard IV hydration. Rescue pain medication will then be given to patients based on the discretion of the treating physician. The infusion preparation will be labeled for the patient with the study number from the computer-generated stratification numbers without any other identifying marks. The study nurse assigned to the patient will administer the study drug.

### Study medications

We will investigate the addition of low-dose ketamine (0.3 mg/kg) in 100 ml normal saline infused over 30 min in addition to standard IV hydration in SCD patients with acute VOC.

### Concomitant medications

Upon arrival and assessment, and within 30 min of patient-physician contact, all patients will receive a standard dose of non-narcotic analgesia of either paracetamol 1 g IV infusion over 30 min or NSAIDS (either lornoxicam 8–16 mg IV or diclofenac 75 mg intramuscular injection).

### Sample size

The sample size was based on the primary efficacy analysis of the mean score for pain, which was tested at a two-sided significance level of 0.05. Based on the assumptions of a standard deviation of 3.41, a mean difference among the groups of 1.5 in the score, and a power of 90%, 220 patients are required. We need to recruit 240 patients since we used the Lan-DeMets O’Brien Fleming approach for interim analysis using a two-sided, asymmetric, beta-spending with nonbinding lower bound at the 0.044 significance level. Additional patients (10%) will be added for a final sample size of 264 patients to compensate for drop-outs during the study.

### Blinding, allocation, and concealment

We intend to blind participants, healthcare providers, and outcomes assessors to treatment allocation. After a patient is considered eligible and written informed consent is obtained, randomization will be performed. An unblinded nurse with no involvement in patient care will randomize the patients via an online, computer-generated sealed envelope program wherein randomization and treatment allocation is concealed. Block size will be used to randomize patients and stratify them according to gender to ensure that the groups are balanced. We will randomize patients in a 1:1 ratio to receive either low-dose ketamine (0.3 mg/kg) in 100 ml normal saline in addition to standard IV hydration or a standard dose of morphine (0.1 mg/kg) in 100 ml normal saline in addition to a standard IV hydration. All enrolled patients will receive the same design of follow-up. We will use the intention-to-treat principles for all analyses.

#### Unblinding

In the case of an adverse event (respiratory depression, severe hypotension, laryngeal spasm, severe allergic reaction, or cardiac arrest), an independent safety monitoring team mandated to identify, evaluate, minimize, and appropriately manage risks will take over. The team consists of qualified independent investigators and clinicians who will unmask treatment allocation and will provide immediate medical care to subjects enrolled in the trial.

### Co-interventions

The ED team will have full independent control of patient management, and any other management shall not be influenced by the allocated intervention. As an example, the ED physician will decide when and what to give as rescue pain medication as usual practice.

Moreover, there will be predefined discharge criteria where patients will be discharged after a minimum of 120 min (2 h) of receiving the study drug if the following criteria are fulfilled: 1) fully awake Glasgow Coma Scale (GCS) score = 15/15; 2) stable vital signs; 3) able to walk independently; and 4) no side effects related to the study drugs.

Conversely, the admission decision will be taken within a maximum of 180 min (3 h) if the following situations occur: 1) patients NPRS remains more than 5; 2) unstable vital signs for any reason; 3) any side effects related to the study drugs; or 4) at the discretion of the ED physician.

### Measures and outcome measures

The primary efficacy outcome will be pain severity scores using the NPRS; the patient will be asked to rate their pain at the initial assessment and then the score will be recorded by the ED nurse every 30 min until a maximum of 180 min, whereupon either a discharge or an admission decision will be made (which may have already been taken based on the abovementioned predefined criteria or discretion of the ED physician). Secondary outcomes include the length of ED stay (defined as the time from the start of the study medication to the discharge from the hospital or admission), the cumulative use of opioids, the hospital admission rate, and the incidence of any drug-related side effects.

### Statistical methods

All data analyses will be carried out according to a pre-established analysis plan. We are planning for complete case analysis and multiple imputations for missing data. All data will be analyzed according to the intention-to-treat principle beginning immediately after randomization.

Demographic and baseline disease characteristics will be summarized with the use of descriptive statistics. Categorical variables will be reported as absolute numbers and percentages. A generalized linear model will be used to compare the two treatment groups. Categorical data and 95% confidence intervals will be calculated by means of the two-by-two table method with the use of log-normal approximation. Continuous variables will be reported as mean ± standard deviation (SD) or median and interquartile range (IQR). Normality will be evaluated using visual histogram evaluation and a Q-Q plot. Between-group differences will be evaluated using the *t* test or Wilcoxon signed rank test, in accordance with normality of the distribution.

Sample size calculation was performed using both PS (Power and Sample Size Calculator, Vr3.04, 2009 http://biostat.mc.vanderbilt.edu/PowerSampleSize) and in the R (R Foundation for Statistical Computing, Vienna, Austria; https://www.R-project.org).

Data analysis will be performed using SPSS (v 16.0 for Windows, IBM) and IBM SPSS Statistics v 25 will be used to calculate the boundaries.

#### Interim analysis

An independent safety committee will perform three interim analyses on information time 25% (70 patients), 50% (140 patients), and 75% (210 patients). Data evaluation at each interim analysis will be based on the alpha spending function concept using a Lan-DeMets O’Brien Fleming approach with the use of a two-sided, asymmetric, beta spending with nonbinding lower bound. For the first interim analysis the efficacy-stopping rule would require an extremely low *P* value (*P* < 0.000015). For the second interim analysis, *P* < 0.0015 will be taken as the efficacy stopping rule. For the third interim analysis, *P* < 0.0081 will be taken as the efficacy stopping rule. Investigators will be kept blinded to the interim analysis results.

## Monitoring

A research coordinator, study investigator, or the study nurse will approach the patient to assess and record primary and secondary outcomes at the designated time intervals. Data are collected by the bedside research nurse upon enrollment on paper case report forms (CRFs). All enrolled patients will receive a random patient identification code. The paper data will be forwarded by the research nurse to the data manager at the end of the 180-h window and will be transcribed electronically and stored digitally, encrypted with a double password, with the hard copy under lock and key. Access to the data-entry system is managed and protected by a personalized username and is password protected.

Missing data will be accounted by complete case analysis and multiple imputations by the data manager, data safety monitoring team, and a statistician.

We will describe serious adverse events as any manifestation, incident, or response to intervention, whether anticipated or not that requires an in-patient admission or extension of an existing hospitalization that results in disability, life-threatening occurrence, or death.

The issue of the safety of the ED patients is a prime concern in this pragmatic randomized clinical trial. Any unexpected safety concerns will be reported to an independent Institution Safety Monitoring Board who will be monitoring the safety of the trial.

## Approval of the study protocol

Prior to the start of the study, the protocol and the informed consent form and other applicable documentation were approved by the Institutional Review Board (IRB-2016-01-042) of our institution. Documentation of Ethics Committee/IRB approvals are required before projects are activated to register and enroll patients.

All signed informed consents will be stored in the emergency department research office. The paper data collection sheets and signed informed consents will be stored in a locked cabinet for safe keeping and made available for trial-related monitoring, audits, and institutional review board and regulatory inspections when required. Federal regulations require research records to be retained for at least 3 years after the completion of the research (45 CFR 46) and will be destroyed at a maximum of 5 years after publication. Only the data manager will have access to the electronic database.

## Discussion

Opioid treatment is currently the mainstay of VOC pain management for SCD patients. However, the long-term use of opioids is limited by the development of opioid tolerance and opioid side effects such as respiratory depression, hypotension, and histamine release. In addition, opioid-induced hyperalgesia highlights the necessity for alternative pain management strategies for these patients. Recently, a better understanding of the pathophysiological mechanisms of VOC pain has led to the development of new classes of drugs that are being tested to provide better pain management for SCD patients with VOC and to overcome the limitations of opioid use.

Activation of NMDA receptors has been found in SCD patients and is suggested to be implicated in opioid-induced hyperalgesia [1]. This finding led to the hypothesis that NMDA antagonists might provide additional benefit for VOC pain control.

Ketamine, a noncompetitive NMDA receptor antagonist, may have the potential to modulate opioid-induced hyperalgesia through impaired sensitization of spinal neurons to nociceptive stimuli, and may therefore reduce neuropathic pain. Lubega et al. [18] performed a randomized controlled trial in pediatric patients with SCD and found that 1 mg/kg of IV ketamine was not inferior to 0.1 mg/kg morphine in terms of maximum improvement in NPRS scores among SCD children with acute severe VOC pain.

Several reports have described successful VOC pain reductions with ketamine infusion [19]. Palm et al. [20] reported a retrospective case series of five SCD patients with prolonged VOC and insufficient pain control with opioid analgesic therapy. The patients were treated with continuous low-dose ketamine infusion up to 5 μg/kg/min. The pain scores were significantly reduced by ketamine infusion, while only one of the five patients reported vivid dreams as an adverse effect of ketamine. These reports show that ketamine provides better pain control as an adjuvant to opioids. However, the small number of patients and the lack of a reliable control group limit such reports.

The predictors of patient response to ketamine and opioids were investigated by Nobrega et al. [21]. They studied a cohort of 84 patients receiving 181 ketamine infusions. The multivariate analysis showed that gender, age group, pain location, and infusion duration independently predicted changes in pain score. They concluded that male patients (*P* = 0.013) of younger age (*P* = 0.018) achieved greater pain reductions than females and older patients.

Published data regarding ketamine for VOC pain management are based on reports with small sample sizes or retrospective designs that limit the generalizability of their findings. In addition, some studies reported the use of ketamine as an adjuvant to opioids. We realized the necessity to provide a direct comparison between low-dose ketamine and opioids as the gold standard framework of a randomized, blinded, controlled trial. Therefore, our study will expand the literature by providing information regarding the safety and efficacy of low-dose ketamine (0.3 mg/kg) in comparison with morphine (0.1 mg/kg) in a randomized, blinded, controlled trial.

This trial will be the largest to date to address this question in an adult population.

### Strengths and limitations

Strengths of this study include blinding; we aim to blind participants, healthcare providers, and outcome assessors to treatment allocation. We also use ketamine, an easily accessible and readily available intervention. A limitation of this study is that it is a single-center study

## Additional files

Additional file 1: SPIRIT checklist. (DOCX 45 kb)

Additional file 2: Ketamine for acute painful crisis in sickle cell disease patients—recognition pathway. (DOCX 115 kb)

Additional file 3: Ketamine for acute painful crisis in sickle cell disease—pain algorithm. (DOCX 176 kb)
