# Supplementary material for: Study protocol for a randomized, blinded, controlled trial of ketamine for acute painful crisis of sickle cell disease
Source: Trials. 2019 May 27;20:286. doi: 10.1186/s13063-019-3394-4 (PMC6537144; doi:10.1186/s13063-019-3394-4)
Supplement: Supplementary file 2 — Ketamine for acute painful crisis in sickle cell disease patients—recognition pathway. (DOCX 115 kb) [file 13063_2019_3394_MOESM2_ESM.docx]

Supplementary # 1

Screened by (Name & Signature): ____________________________

Date: _______________________ Time: _____________________

Met all eligibility criteria?

Stick Patient Label here

**Ketamine for Acute Painful Crisis in Sickle Cell Disease**

**Patients Recognition Pathway**

Send Randomization slip to the Pharmacy

Met the criteria & can be enrolled in the study

NO

YES

YES

Obtain Consent

EXCLUDED

NO

| Pregnancy or breast feeding  Altered mental status  Body mass index greater than 40 kg/m2  With significant neurological disease  Seizures  Acute head or eye injury  With high intra-cranial tension  With known psychiatric disorders  With significant cardiac disease or arrhythmias  With significant pulmonary diseases other than acute chest syndrome | With significant renal disease (BUN/creatinine ration ≤ 25)  With significant hepatic disease (Child Pugh Class B or C)  With significant endocrine disease  Known allergy to phencyclidine derivatives, ketamine or morphine  Sepsis or septic shock  Requiring circulatory or mechanical ventilatory support  Alcohol or drug abuse  Patients with chronic pain status unrelated to SCD  Patients receiving anti-convulsant or anti-psychiatric medications  Patients with communication barriers. |
| --- | --- |

Exclusion Criteria Checklist

**Eligibility Criteria**:

- Patient is ≥ 18 years of age on visit to ED
- With known diagnosis of SCD based on Sickle Cell tests and hemoglobin electrophoresis
- Acute onset of painful crisis (defined as having an onset within 7 days)
- Pain score of >5 (NPRS= NUMERICAL PAIN RATING SCORE; VAS= VISUAL ACUITY SCORE)
