# Supplementary material for: Study protocol for a randomized, blinded, controlled trial of ketamine for acute painful crisis of sickle cell disease
Source: Trials. 2019 May 27;20:286. doi: 10.1186/s13063-019-3394-4 (PMC6537144; doi:10.1186/s13063-019-3394-4)
Supplement: Supplementary file 3 — Ketamine for acute painful crisis in sickle cell disease—pain algorithm. (DOCX 176 kb) [file 13063_2019_3394_MOESM3_ESM.docx]

Manage pain in the fast track clinic

- Treat specific complaint,
- If pain is resolved, discharge home.

**NO**

**YES**

**Should be assessed within 30 minutes upon arrival**

**Numerical Pain Rating Score (NPRS) above 5?**

Consider transfer to monitoring bed

- **Patients Recognition Pathway**

**(Screen for possible study inclusion**

**Was the pain resolved?**

**If pain is resolved, discharge home.**

Discharge Criteria where patients after a minimum of 120 minutes (2 hours) of treatment will be discharged if the following criteria are fulfilled:

- Fully awake with a GCS= 15/15
- Stable vital signs
- Able to walk independently

Re-assess for pain, pulse oximetry, and sedation, using a validated scales as Numerical Pain Rating Score (NPRS) & Richmond Agitation Sedation Scale (RASS), at 30, 60, 90, 120 and 180 minutes.

•Re-administer analgesic doses every 30 minutes until pain relief is obtained

1. All patients, will receive a standard dose of non-narcotic analgesia, either paracetamol or NSAID
2. Pain score will be measured at 0 and 30 minutes, after which consent procedure and randomization will be initiated for patients meeting inclusion criteria (See Patient recognition pathway)
3. Patients who will be randomized to the intervention group will receive low-dose ketamine 0.3 mg/kg in 100 ml normal saline infused over 30 minutes in addition to a standard IV hydration, Then, rescue pain medication will be given to patients based on the treating physician discretion’s while patients who will be randomized to the control group will receive the standard dose of morphine (0.1 mg/kg) in 100 ml normal saline infused over 30 minutes in addition to a standard IV hydration.
4. Rescue pain medication will be given to patients based on the treating physician’s discretion.

- May require IV fluid bolus 10-20 mg/ kg. Avoid excess fluids to reduce risk of chest crisis.
- Patients may require blood transfusion – discuss with Haematology fellow.
- Early ICU review and respiratory support if concern for acute chest crisis.

Investigations:

- CBC including reticulocyte count
- RFTs and LFTs
- CXR if respiratory symptoms
- Other imaging required.
- Other investigations as needed

**For minimal or no change in pain, refer to Internal Medicine for admission.**

**Admission decision to be taken within a maximum of 180 minutes (3 hours) if:**

**•Patients pain score still more than 5 (NPRS).**

**•Unstable vital signs for any reason**

**•ED physician discretion**

**Abbreviations:**

(SCD) Sickle Cell Disease; (VOC) Vaso occlusive Crisis; (NPRS) Numerical Pain Rating Score; (CBC) complete blood count; (RFT) Renal function test; (LFT) Liver function test; (CXR) Chest x-ray; (IV) Intravenous; (NSAID) Non-steroidal anti-inflammatory drug; (GCS) Glasgow Coma Score

*Supplementary # 2*
